# Multi-Quantifying Maxillofacial Traits via a Demographic Parity-Based AI Model

**DOI:** 10.34133/bmef.0054

**Published:** 2024-08-13

**Authors:** Mengru Shi, Zhuohong Gong, Peisheng Zeng, Dawei Xiang, Gengbin Cai, Hengyi Liu, Shijie Chen, Runheng Liu, Zhuofan Chen, Xinchun Zhang, Zetao Chen

**Affiliations:** Hospital of Stomatology, Guanghua School of Stomatology, Guangdong Provincial Key Laboratory of Stomatology, Sun Yat-sen University, Guangzhou 510055, China.

## Abstract

**Objective and Impact Statement:** The multi-quantification of the distinct individualized maxillofacial traits, that is, quantifying multiple indices, is vital for diagnosis, decision-making, and prognosis of the maxillofacial surgery. **Introduction:** While the discrete and demographically disproportionate distributions of the multiple indices restrict the generalization ability of artificial intelligence (AI)-based automatic analysis, this study presents a demographic-parity strategy for AI-based multi-quantification. **Methods:** In the aesthetic-concerning maxillary alveolar basal bone, which requires quantifying a total of 9 indices from length and width dimensional, this study collected a total of 4,000 cone-beam computed tomography (CBCT) sagittal images, and developed a deep learning model composed of a backbone and multiple regression heads with fully shared parameters to intelligently predict these quantitative metrics. Through auditing of the primary generalization result, the sensitive attribute was identified and the dataset was subdivided to train new submodels. Then, submodels trained from respective subsets were ensembled for final generalization. **Results:** The primary generalization result showed that the AI model underperformed in quantifying major basal bone indices. The sex factor was proved to be the sensitive attribute. The final model was ensembled by the male and female submodels, which yielded equal performance between genders, low error, high consistency, satisfying correlation coefficient, and highly focused attention. The ensemble model exhibited high similarity to clinicians with minor processing time. **Conclusion:** This work validates that the demographic parity strategy enables the AI algorithm with greater model generalization ability, even for the highly variable traits, which benefits for the appearance-concerning maxillofacial surgery.

## Introduction

The maxillofacial appearance is composed of various maxillofacial traits with prominent individual variances, which make each person recognizable [1,2] . Confronting with such highly variable maxillofacial traits, the oral and maxillofacial surgery, which aims to rehabilitate the compromised traits toward a more aesthetic appearance, meets with the challenge of comprehensive and repeatable representation of the complex traits. Under these circumstances, the quantification rather than the classification of these traits becomes the optimized method to obtain precise facial analysis. For example, the cephalometric measurements of the SNA (sella-nasion to A point), SNB (sella-nasion to B point), and ANB (difference between SNA and SNB) angles diagnose the type of dental-maxillofacial deformities [3]. The quantifications of the horizontal and vertical dimensions of the alveolar socket suggest the bone augmentation selection during the oral implantology [4,5]. Hence, the quantification of the highly individualized maxillofacial traits is important for diagnosis, decision-making, and prognosis of the appearance-changing treatments.

The quantification of these maxillofacial traits is never an easy task. The individualized appearance generates a large number of traits, and the traditional manual measurement of these massive traits is time-consuming and error-prone. With the development of artificial intelligence (AI), this automatic algorithm acquires the ability to abstract the unified feature and function approximation to the real world, which has succeeded in facial recognition [6], anatomic detection [7], and dental disease classification [8] in an automatic, effective, and objective manner [9]. For example, the automatic measurement of the maxillofacial traits like the ANB angle provides guidance for diagnosis of skeletal malformities [10]. Further, as for the quantitative task, we have succeeded in quantifying single-variable maxillofacial index (i.e., the root inclination angle) via an end-to-end convolutional neural network (CNN) [11]. Hence, there is a good chance that the CNN-based algorithm is capable of quantifying the individualized traits.

However, the comprehensive representation of the maxillofacial traits requires multiple rather than single indices for individualized analysis. For instance, the maxillary alveolar basal bone requires 4 length and 5 width indices to determine the extent of alveolar bone and the degree of primary stability for dental implant placement [12]. A possible solution for automatic multi-quantification is to simultaneously train numbers of networks that output the corresponding single index. But this is not clinically feasible for the redundancy of network storage, dozens of computation burdens, and prolonged operation time. These require AI algorithms to come up with the generalization ability to compute all quantitative maxillofacial traits at one time.

**Fig. 1.**
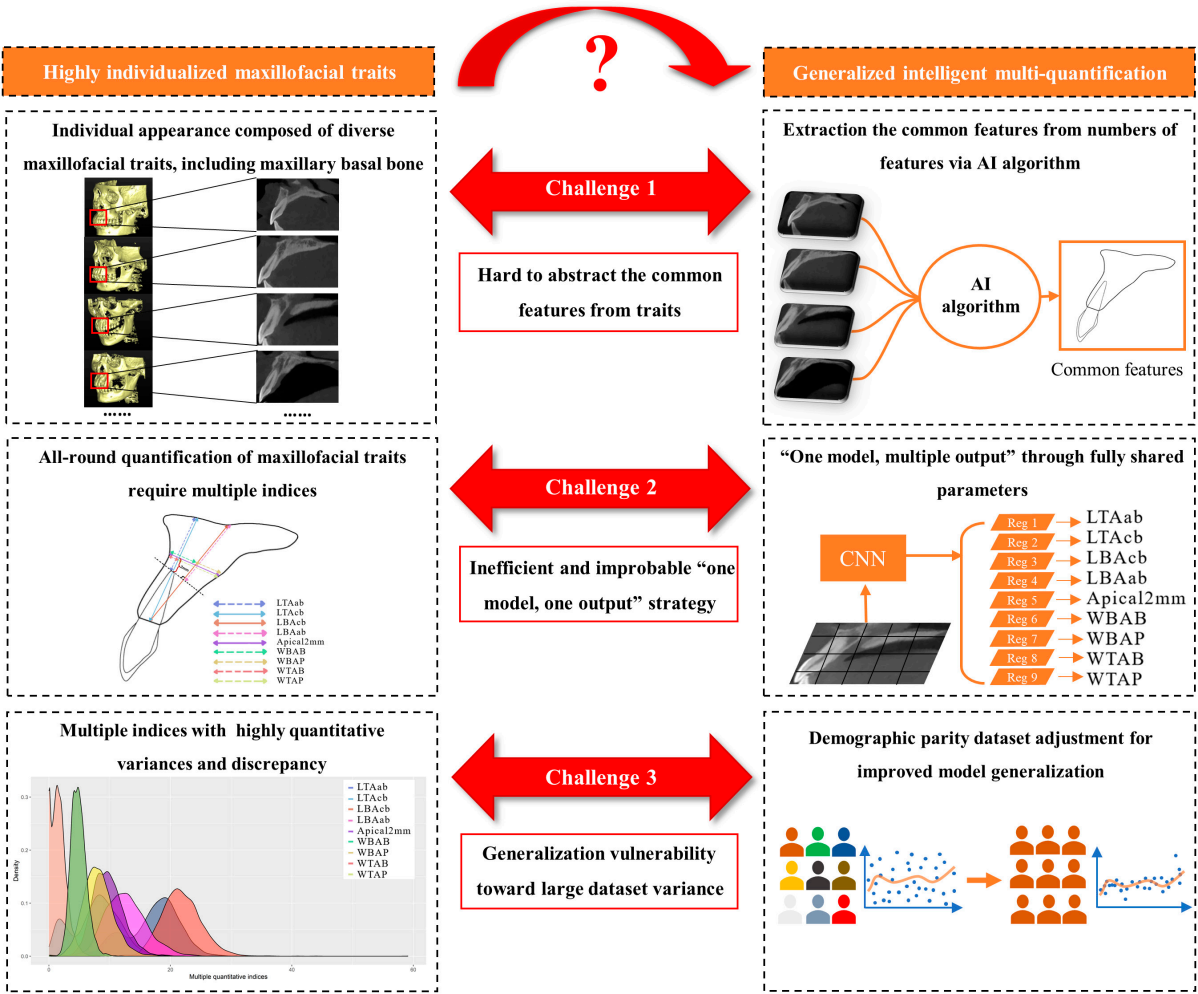
The schematic of the challenges in highly variable maxillofacial traits versus generalized intelligent multi-quantifications. The first challenge is the difficulties to extract the common features from the highly diverse traits. The second challenge is the inefficient "one model, one output" strategy toward multi-quantifications. The third challenge is that the discrete multiple quantitative indices lead to insufficient model generalization ability.

Unfortunately, the generalization ability is restricted due to the highly discrete distribution of the multi-quantifications among populations from the demographic-related individualized variance of these maxillofacial traits [13]. It is assumed that under these demographic-skewed datasets, the algorithms find the shortcuts to learn the patterns of specific subpopulations rather than considering the important sensitive attributes that the algorithms should not discriminate against, and as a result, the model underserves certain subpopulations [14] (Fig. 1). Interestingly, accumulated evidence demonstrates that the parity of the demographics within the dataset has the potential to advance generalization by mitigating bias [15]. By utilizing the sensitive demographic attribute to demarcate the shifted dataset, the individuals in the renewed dataset possess higher similarity [16]. This demographic parity strategy is expected to represent the genius patterns of specific subpopulations and improve the robustness of AI algorithm even in highly discrete multi-quantifications [17].

**Fig. 2.**
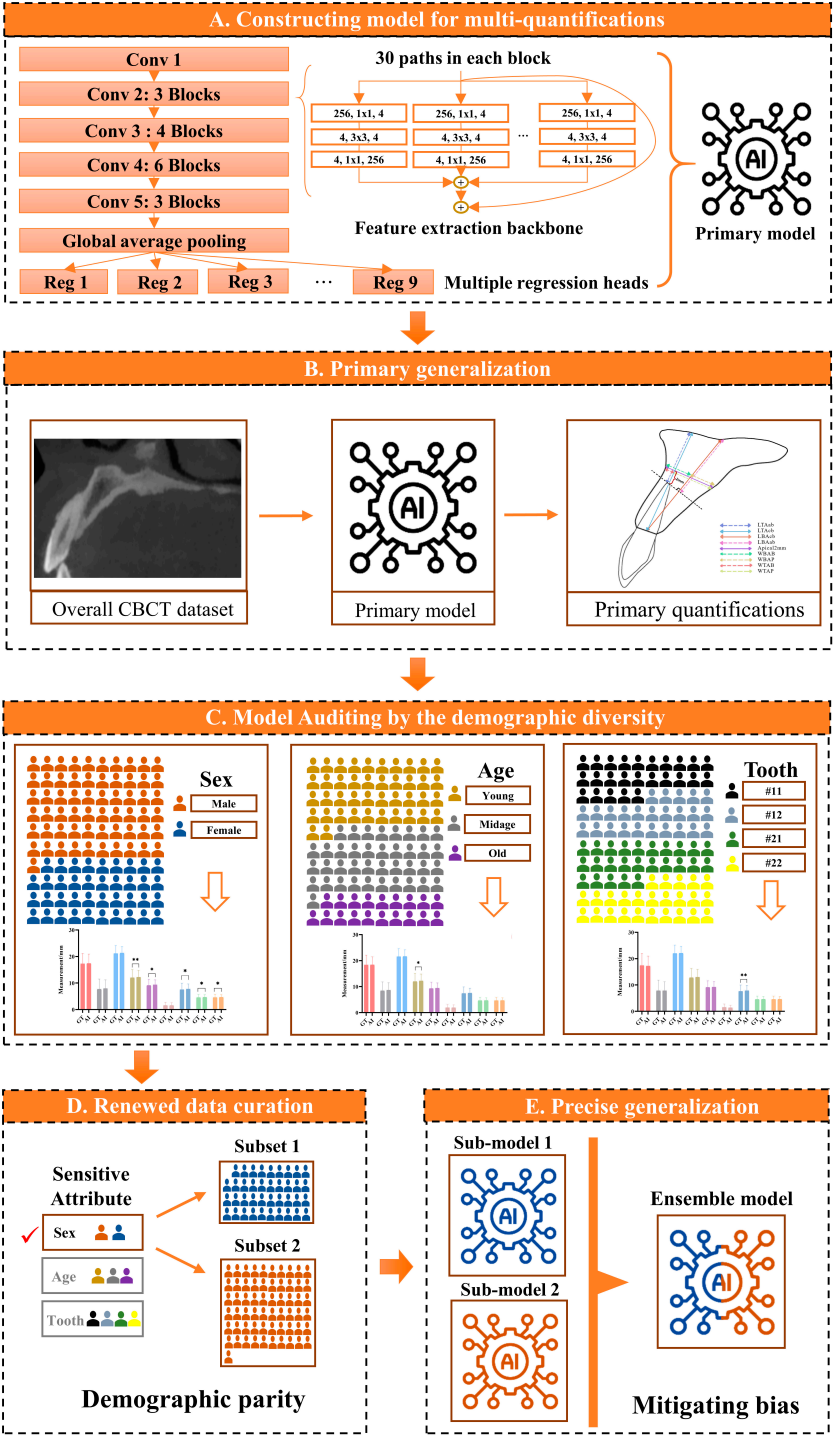
The schematic of the workflow of the deep learning-based multi-quantifications of the variable maxillary basal bone via the demographic parity-based strategy. (A) Construction of multi-quantification model with a feature extraction backbone and multiple regression heads with fully shared parameters. (B) The primary generalization result is acquired based on the overall cone-beam computer tomography (CBCT) dataset, which serves as the data sources to investigate the bias that hinders the further generalization. (C) The model auditing is performed by investigating the potential bias-related demographic diversity, including the sex, age, and tooth, where the manual ground truth (GT) measurements are compared with the model predictions (AI). (D) Based on the sensitive attribute that found during the model auditing, the data curation protocol is renewed to subdivide the overall dataset into different subsets with internal demographic parity. (E) The submodels that trained and validated on the respective subsets are ensembled to mitigate the bias and realize the precise generalization toward the whole populations.

To validate the demographic parity strategy toward the generalization to diverse maxillofacial traits, this study developed a deep learning model for automatic multi-quantifications of the maxillary alveolar basal bone from cone-beam computed tomography (CBCT). The algorithm consisted of a CNN backbone for feature extraction and several regression heads with shared parameters. The proposed deep learning model was directly applied to the large diverse dataset for primary generalization. Then, the model auditing found the sensitive demographic factors. The sensitive attribute was applied to renew the dataset and models. The precise generalization was performed by assembling the renewed models (Fig. 2). The hypothesis of this study is that the demographic-parity strategy based AI model has high generalization ability and yields clinically trusted multi-quantifications in comparison to clinicians.

## Results

### Study design and data collections

This study collected anonymous CBCT from 1,000 patients (mean age: 36.15 ± 13.09 years, 388 male and 612 female) from the Hospital of Stomatology of Sun Yat-sen University, through which 4,000 images of mid-facial sagittal sections were acquired, noted as #11, #12, #21, and #22 for right and left central and lateral maxillary incisors, respectively. A total of 36,000 indices (9 indices per image) of maxillary alveolar basal bone were measured. The overall dataset was randomly assigned into the training set, validation set, and test set to 6:2:2 in a patient-wise manner (Table S1).

### Construction of the end-to-end multi-quantification model

This study built an end-to-end multi-quantification model with a CNN backbone and 9 parallel regression heads with fully shared parameters (Fig. 2). Among all the backbone candidates (Resnet50, Resnet101, Resnext50, Resnext101, Wide-Resnet50, and Wide-Resnet101), the ResNeXt-50 backbone yielded the lowest mean square error (MSE, 2.236) in the validation set (Fig. S1). Based on the backbone of ResNeXt-50, the hyperparameter setting of the learning rate of 0.0005 and the batch size of 4 exhibited the minimum error. The pretraining and data augmentation methods were proved as the optimized training strategy (Fig. S2). The loss convergence plot indicated that the loss of the model became stable at approximately 200 epochs (Fig. S3).

### Evaluation of the primary generalization performance of multi-quantification on the overall dataset

The model was performed on the overall anonymous dataset to acquire the primary generalization performance. Compared to the manual ground truth (GT) measurements of maxillary basal bone in the test set, the model predictions by the primary model (AI) were slightly larger in major indices, except for the index of width from buccal counter to the tooth axis (WTAB). The mean differences between GT and AI measurements of all indices ranged from −0.03 to 0.21 mm. However, a large proportion (7/9) of maxillary basal bone indices exhibited statistical differences between the AI-GT measurements, including the alveolar crest to the basal bone along the tooth axis (LTAcb), the apex to the basal bone along the tooth axis (LTAab), the apex to the basal bone along the bone axis (LBAab), bucco-palatal width at 2 mm apical to apex (apical2mm), width from palatal counter to the tooth axis (WTAP), width from buccal counter to the bone axis (WBAB), and width from palatal counter to the bone axis (WBAP), while only the alveolar crest to the basal bone along the bone axis (LBAcb) and width from buccal counter to the tooth axis (WTAB) showed no significant difference (Table 1).

**Table 1.** The performance of the multi-quantification model on the overall maxillary basal bone dataset. The *P* value was calculated through paired-sample Wilcoxon signed-rank test: *P < 0.05, **P < 0.01, ***P < 0.001.

Metrics	LTAcb	LTAab	LBAcb	LBAab	apical2mm	WTAB	WTAP	WBAB	WBAP
Ground truth	17.58 ± 4.05	7.91 ± 3.81	21.82 ± 3.19	12.57 ± 3.31	9.69 ± 2.44	1.71 ± 1.28	7.98 ± 2.49	4.85 ± 1.22	4.85 ± 1.22
Model predictions	17.79 ± 3.42	8.13 ± 3.15	21.95 ± 2.64	12.69 ± 2.75	9.83 ± 2.06	1.68 ± 1.07	8.15 ± 2.01	4.91 ± 1.03	4.92 ± 1.03
Difference	0.21 ± 2.62	0.21 ± 2.46	0.13 ± 1.84	0.12 ± 1.98	0.14 ± 1.63	−0.03 ± 0.71	0.18 ± 1.74	0.07 ± 0.82	0.07 ± 0.82
*P* value	0.028*	0.027*	0.122	0.026*	0.022*	0.487	0.009**	0.031*	0.021*

### Model auditing to determine the sensitive attribute

**Fig. 3.**
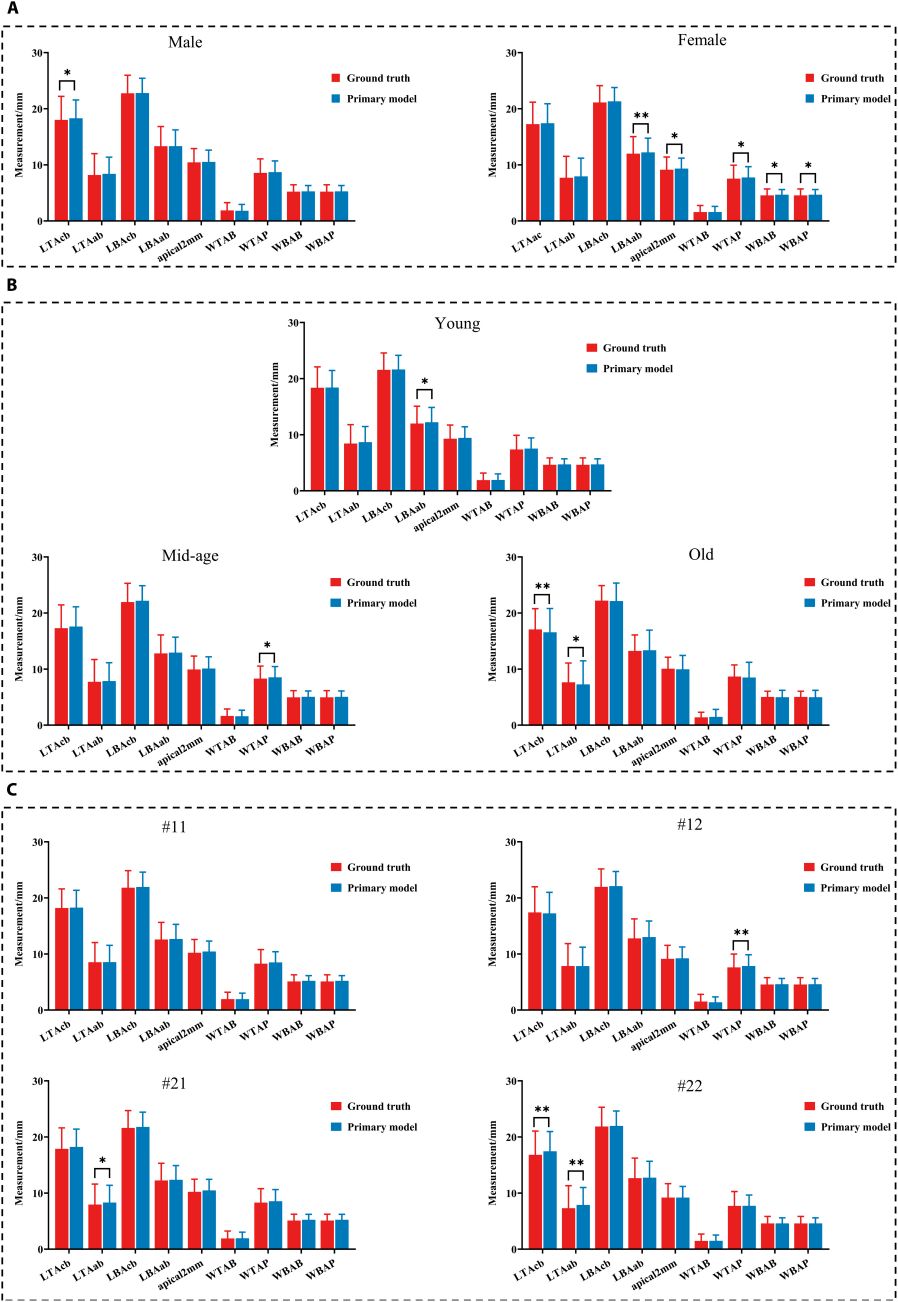
The model auditing to determine the sensitive demographic attribute. The potential bias-related demographic diversities include sex (A), age (B), and tooth (C), which are compared between the manual GT and the result of primary model. The *P* value was calculated through paired-sample Wilcoxon signed-rank test. *P < 0.05, **P < 0.01, ***P < 0.001.

The model auditing included the investigations into the influence of population diversity on the primary generalization performance, including the sex, age, and tooth site, to determine the sensitive attribute (Fig. 2C). In the male individuals from the test set, all indices but one (LTAcb) had no significant AI-GT difference. On the contrary, numbers of indices (LBAab, apical2mm, WTAP, WBAB, and WBAP) were significantly different between AI and GT in the female individuals. From the aspect of age and teeth, there are a few different indices between different subsets (Fig. 3). The model auditing implied that sex shall be the sensitive attribute that hazards the generalization.

### Demographic parity strategy to alleviate the underperformance in generalization

**Fig. 4.**
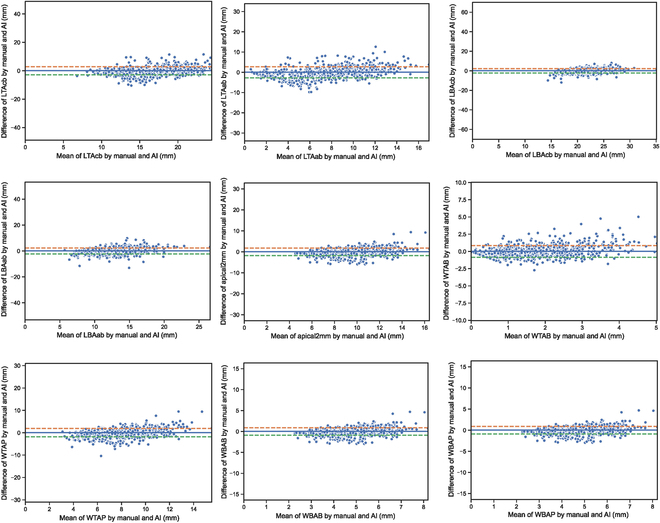
The Bland–Altman plots of multi-quantifications of the maxillary basal bone via the ensembled model with demographic parity strategy.

**Fig. 5.**
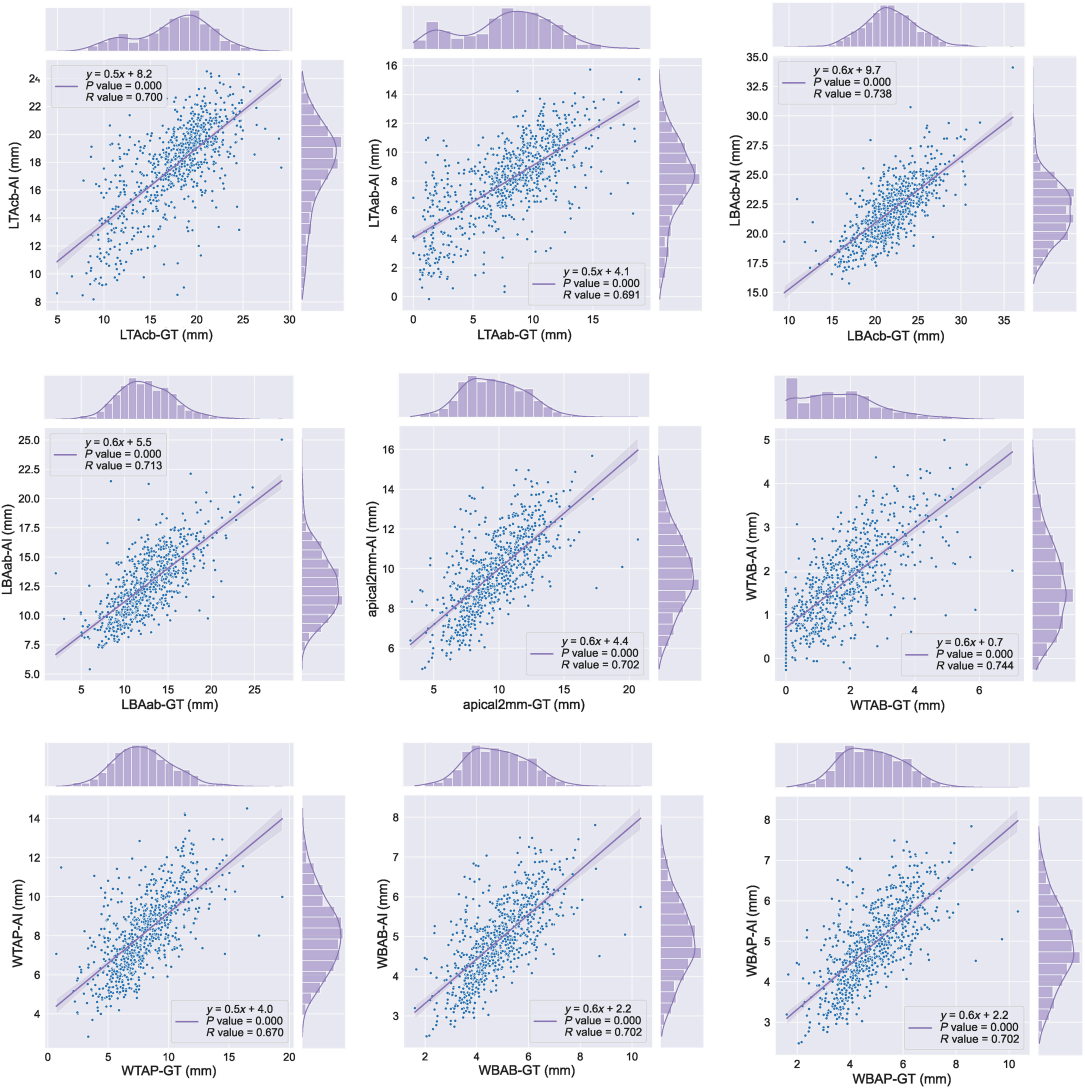
The scatterplots of multi-quantifications of the maxillary basal bone via the ensembled model with demographic parity strategy.

**Fig. 6.**
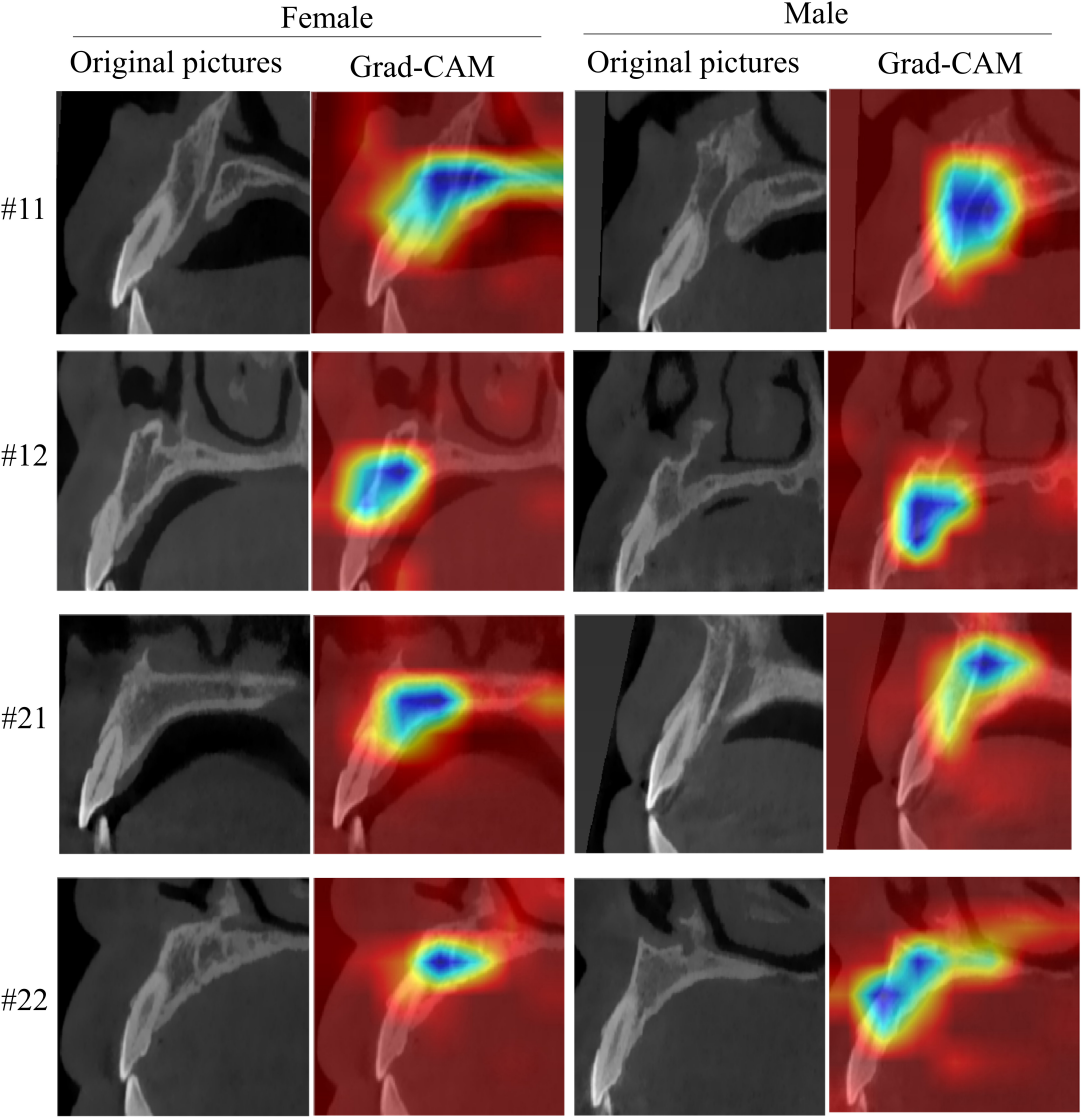
The Grad-CAM visualization heatmaps of the ensembled multi-quantification model.

Guided by the demographic parity strategy and the sensitive attribute (i.e., sex), the dataset was subdivided into male- and female-only subsets (Fig. 2D). Two independent submodels were trained in the female and male subsets. For the female-only submodel, all of the indices showed no significant GT-AI difference, accompanying with a high correlation (*r* = 0.706 to 0.784) and interclass correlation coefficient (intraclass correlation coefficient [ICC] = 0.683 to 0.766). Likewise, the male-only submodel exhibited only one different index (LTAab, *P* = 0.013), accompanied by a slightly lower correlation (*r* = 0.549 to 0.697) and consistency (ICC = 0.487 to 0.663). The predictions of male- and female-only submodels were ensembled. The ensemble result showed considerable progress in alleviating the biased predictions of AI, with the significant AI-GT difference falling from 7 indices to 1 index (i.e., WTAP). From the perspective of consistency, the ICC of all indices between the GT and AI measurements was >0.50, with the lowest and highest ICCs of 0.645 (WTAP) and 0.719 (WTAB) (Fig. 4). Similarly, all the AI measurements depicted strong correlations with the GT measurements (Fig. 5), with the Pearson correlation coefficients ranging from 0.670 to 0.744 (Table 2). As depicted by the visualization heatmaps through the Grad-CAM, the ensemble model accurately focused on the region of the maxillary basal bone (Fig. 6).

**Table 2.** The performance of single as well as ensemble models on quantifying the maxillary basal bone indices based on the demographic parity-based strategy. The *P* value was calculated through paired-sample Wilcoxon signed-rank test: *P < 0.05, **P < 0.01, ***P < 0.001.

Metrics	LTAcb	LTAab	LBAcb	LBAab	apical2mm	WTAB	WTAP	WBAB	WBAP
A. Male-only model									
Ground truth	18.01 ± 4.19	8.19 ± 3.81	22.76 ± 3.22	13.33 ± 3.51	10.45 ± 2.45	1.88 ± 1.39	8.56 ± 2.50	5.22 ± 1.23	5.22 ± 1.23
Model predictions	18.30 ± 2.81	8.56 ± 2.36	22.80 ± 2.46	13.48 ± 2.77	10.56 ± 1.89	1.84 ± 1.01	8.71 ± 1.81	5.28 ± 0.94	5.28 ± 0.94
Difference	−0.29 ± 3.39	−0.37 ± 3.20	−0.04 ± 2.33	−0.15 ± 2.75	−0.11 ± 1.93	0.04 ± 1.00	−0.15 ± 2.07	−0.05 ± 0.97	−0.05 ± 0.97
MAE	2.58	2.54	1.90	2.12	1.41	0.73	1.50	0.70	0.71
*P* value	0.070	0.013*	0.982	0.429	0.313	0.485	0.175	0.356	0.343
*r* value	0.593	0.549	0.658	0.639	0.631	0.697	0.581	0.629	0.630
ICC	0.547	0.487	0.636	0.622	0.663	0.663	0.552	0.608	0.609
B. Female-only model									
Ground truth	17.26 ± 3.91	7.71 ± 3.80	21.12 ± 3.00	12.00 ± 3.03	9.13 ± 2.27	1.59 ± 1.17	7.54 ± 2.40	4.56 ± 1.14	4.56 ± 1.14
Model predictions	17.24 ± 3.27	7.64 ± 2.98	21.26 ± 2.19	12.03 ± 2.35	9.26 ± 1.80	1.57 ± 0.94	7.69 ± 1.86	4.63 ± 0.90	4.63 ± 0.90
Difference	0.22 ± 2.48	0.71 ± 2.34	−0.14 ± 1.92	−0.03 ± 1.97	−0.14 ± 1.60	0.02 ± 0.73	−0.15 ± 1.71	−0.68 ± 0.80	−0.67 ± 0.80
MAE	1.78	1.68	1.45	1.44	1.13	0.55	1.21	0.57	0.757
*P* value	0.916	0.498	0.221	0.377	0.239	0.429	0.138	0.236	0.255
*r* value	0.776	0.782	0.769	0.760	0.712	0.784	0.706	0.712	0.712
ICC	0.764	0.760	0.732	0.736	0.692	0.766	0.683	0.692	0.692
C. Ensemble model									
Ground truth	17.58 ± 4.05	7.91 ± 3.81	21.82 ± 3.19	12.57 ± 3.31	9.69 ± 2.44	1.71 ± 1.28	7.98 ± 2.49	4.85 ± 1.22	4.85 ± 1.22
Model predictions	17.69 ± 3.12	8.03 ± 2.77	21.92 ± 2.43	12.65 ± 2.64	9.82 ± 1.95	1.69 ± 0.98	8.13 ± 1.91	4.91 ± 0.97	4.91 ± 0.97
Difference	−0.11 ± 2.90	−0.12 ± 2.76	−0.10 ± 2.16	−0.08 ± 2.33	−0.13 ± 1.75	0.03 ± 0.85	−0.15 ± 1.87	−0.06 ± 0.88	−0.06 ± 0.88
MAE	2.12	2.04	1.65	1.73	1.25	0.63	1.33	0.62	0.63
*P* value	0.237	0.205	0.383	0.114	0.280	0.043*	0.127	0.131
*r* value	0.700	0.691	0.738	0.713	0.702	0.744	0.670	0.702	0.702
ICC	0.677	0.657	0.711	0.695	0.684	0.719	0.645	0.683	0.683

### Comparison between clinicians and multi-quantification model

The quantification performance of multi-quantifying maxillary basal bone was compared among junior clinicians, senior clinicians, GT specialist, and the ensemble model. The ensemble model showed satisfying inter-rater reliability between either the junior (ICC: 0.572 to 0.855) or the senior dentists (ICC: 0.516 to 0.858). From the aspect of efficacy, the ensemble model spent remarkably less time on the measurements compared to the dentists (Table 3).

**Table 3.** The measurement reliability (A) and efficacy (B) among clinicians from different levels (junior clinician, senior clinician, and ground truth [GT] specialist) and deep learning model (AI)

A. Measurement reliability									
ICC	LTAcb	LTAab	LBAcb	LBAab	apical2mm	WTAB	WTAP	WBAB	WBAP
Junior clinician vs. GT	0.763	0.710	0.806	0.695	0.520	0.817	0.522	0.520	0.520
Senior clinician vs. GT	0.925	0.893	0.816	0.634	0.516	0.877	0.482	0.516	0.516
Junior clinician vs. AI	0.676	0.643	0.634	0.572	0.625	0.855	0.651	0.625	0.625
Senior clinician vs. AI	0.677	0.620	0.660	0.599	0.633	0.858	0.651	0.516	0.633
B. Efficacy									
Predictor					Time per image/s				
Junior clinician					199.5				
Senior clinician					157				
AI					0.007				

## Discussion

In this study, we constructed a model with a ResNeXt-50 backbone as well as parallel regression heads and successfully applied this model in the scenario of maxillary alveolar basal bone. The primary generalization result reflected the underperformance in the demographic-skewed dataset. The model auditing revealed the sensitive attribute in this task (i.e., sex), and the renewed data curation protocol enabled equal performance between sexes. Guided by the demographic parity-based strategy, the results of the ensemble model were highly correlated and consistent with the clinicians. Within the limit of our knowledge, this study was the first attempt to automatic multi-quantitative analysis of the variable maxillofacial traits with decent generalization ability.

The end-to-end multi-quantification model in this study succeeded in extracting the high-order common features to generalize to major indices of maxillary basal bone. Similar studies corroborated that the end-to-end CNN model yielded trusted performance in quantifications. Lin and colleagues [11] constructed an end-to-end model based on the ResNeXt-101 backbone to determine the sagittal root inclination from CBCT. The mean absolute error (MAE) of the predicted root inclination was 1.90 to 2.18 and ICCs were reasonable between the model and observers. Moriyama et al.’s [18] work adopted a direct MapReduce-like network to estimate the periodontal pocket depth from oral images and achieved a quantification accuracy of 91.7%. By comparison of the aforementioned studies, the final ensembled model not only extended from single-quantification to multi-quantification (a total of 9 indices) but also obtained an inferior error range with the MAE of 0.62 to 2.12. The model performed at the senior clinicians’ level in most indices, except one highly discrete index (i.e., WTAP), which might result from the inherent difficulty difference between each index. More convincingly, the Grad-CAM heatmap exposed the focused regions of interest on the maxillary basal bone, either in male or female subsets.

The algorithmic generalization ability in the condition of multiple discrete quantitative indices is attained by the following 3 contributors. One important contributor is the ResNeXt-50 backbone, which facilitates the end-to-end representation of high-dimensional features with heterogeneous scales and makes full use of the combination of global and local features. The potential reasons may fall in the “split-transform-merge” architecture, that is, splitting the input feature and transforming it into 32 different batches, and finally merging the processing feature of the same topology [19]. On the other hand, the residual block architecture is equipped with the inherent generalization ability, guaranteeing its performance in the unseen targets. Studies had it that the ResNeXt-based backbones were also capable in tasks for variable structures like chromosome [20], thoracic organs [21], molar incisor hypomineralization [22], and forth. Second, the network design of multiple regression heads with fully shared parameters empowers the multi-quantification outputs at one time as well as the translationally invariant duplication of the high-order features to all the subtasks [23]. However, it should be mentioned that the auditing of the primary generalization performance showed significant difference in the performance of different sex subgroups in the major indices. This emphasizes that the dataset quality is essential in improving the model generalization ability.

The further key attribute for improving the dataset quality and model generalization ability to the multi-quantifications of the variable traits is the demographic parity strategy. Aiming at achieving the goal of “demographic parity”, the demographic parity-based strategy finds the sensitive demographic attribute that hinders the model generalization and applies this demographic attribute for dataset subdivision and model ensemble. In this study, we proposed a demographic parity-based strategy to fit all the subpopulations without changing the model framework. The underlying explanations were that there was a natural gender difference in the size, shape, and development patterns of maxillary facial appearances [24]. As for the maxillary basal bone dimension, Zhang and colleagues [25] found that the basal bone width was larger in male than in female, reflecting the rationale for why sex is the sensitive attribute in the scenario of quantifying the maxillary basal bone. The gender discrimination might be because that the model obtains the latent gender information by extracting potential high-dimensional features [26]. Hence, we disentangled the dataset into male- and female-only subsets to minimize the intra-population dataset bias for better generalizing the sex attribute in the representation space. The demographic parity-based strategy is also expected to be applicable in complex situations with large intra-group variances, including the sex-related caner, age-related anatomical degradation, region-based diseases, and so on. Some studies believed that the attempts to generalization may result in lower model accuracy [14]. Wu et al. [27] used the fair-prune method to improve the algorithmic fairness in skin disease diagnosis model, but the accuracy decreased. To our delight, our ensemble model reduced algorithmic unfairness without a significant drop in accuracy (Table 2), which may because the reassigned subset had more similar features and was more conducive to the fitting of the neural network.

This study provides valuable application potentials (Fig. S4). In the clinical aspect, this study successfully achieved automatic multi-quantitative evaluation of maxillary alveolar basal bone, which will be used in different specialties such as oral implantology, orthodontics, and maxillofacial surgery. It can improve diagnostic accuracy, shorten treatment planning time, guide individualized treatment design, and simplify the long-term follow-up for patients. In the scientific research aspect, this study proposed the demographic parity-based strategy that has great potential for transforming to other representative traits that exhibit large population variances, such as sagittal skeletal type analysis, dental aesthetics analysis, and so on. This study may help promote smart healthcare and provide potential avenues for large-scale multicenter clinical research in the future. Further consideration shall be paid to the large-scale application of these methods to the real-world data. For one thing, the data noise generated by the multi-center dataset is dozens of times greater than in the single one, and therefore, the auditing of the sensitive demographic attribute requires more in-depth analysis. For another, the demographic parity-based ensembled models were still inferior in certain indices (i.e., WTAP). This calls for updated model architecture and training strategies toward stronger robustness and generalization to ensure greater accuracy and wider clinical applicability.

### Conclusion

This study validated the feasibility of applying the end-to-end CNN model to the unified multi-quantifications and the benefits of the demographic parity strategy toward the model generalization under the task of multi-quantification of the variable maxillary basal bone with large discrepancy. The demographic parity strategy serves as a potentially vital approach to promote the AI algorithms with advanced generalization ability toward highly discrete demographic settings and clinical-trusted multi-quantification accuracy.

## Materials and Methods

### Subjects and data collection

This study conforms to the Helsinki Declaration and received ethic approval from the Ethic Committee of the Hospital of Stomatology, Sun Yat-sen University (KQEC-2020-29-06). This study is in accordance with TRIPOD-AI reporting guidelines [28]. All enrolled patients signed informed consent prior to inclusion criteria and were informed of the relevant content. The inclusion requirements were as follows: (a) adults aged 18 years or older and (b) the presence of bilateral maxillary central and lateral incisors. The exclusion criteria for subjects were as follows: (a) the included teeth have periapical lesions, severe periodontal disease, or alveolar bone resorption; (b) the included teeth are severely discrete or distorted; and (c) the included teeth are undergoing orthodontic or dental treatment.

CBCT images were acquired by a technological device (NewTom VG; QR s.r.l., Verona, Italy) at a tube voltage of 110 kV, 5 mA, with a field of view of 15 cm × 12 cm. The CBCT images were first imported into coDiagnostiX software (version 9.12; Dentalwings, Montreal, Canada). The jaw plane was then adjusted to be parallel to horizontal plane, and the dental arch curve was drawn at cementoenamel junction (CEJ) level. Finally, by passing through the middle of the teeth, mid-facial sagittal sections of 4 anterior teeth were acquired in TIFF format [29].

The 4 length and 5 width indices of maxillary alveolar basal bone were measured by a specialist with more than 10 years of dental implant clinical experience in Adobe Illustrator software (version 4.0, Adobe Systems Inc., California, USA) as the gold standard reference. The intra-rater ICC of the specialist ranges from 0.885 to 0.977, suggesting good reliability. The lengths of the maxillary basal bone include LTAcb, LTAab, LBAcb, and LBAab. Maxillary basal bone widths include apical2mm, WTAB, WTAP, WBAB, and WBAP (Fig. 1).

### Model design and construction

This study aimed to build an end-to-end multi-quantification model with one major CNN backbone for basal bone index prediction, followed by multiple regression heads for multiple index quantifications. The multiple regression heads shared the same backbone (fully shared parameters) and possessed their own task-specific parameters in the regression process (Fig. 2). Based on previous evidence, the residual blocks were favored for the quantification task of dental structure [11]. In this case, this study adopted Resnet50, Resnet101, Resnext50, Resnext101, Wide-Resnet50, and Wide-Resnet101 as backbones. The optimal hyperparameter settings were examined in the validation process, including the learning rate (0.0005, 0.0003, 0.0001, 0.00005, 0.00001), the batch size (2, 4, 6), and the pretraining and data augmentation (flip) choice. The MSE was set as the major evaluation metric to select the best-fitting network backbone and hyperparameter setting.

### Performance of the multi-quantification model on the overall dataset

This study primarily applied the multiple quantification model to the overall CBCT dataset and yielded the primary baseline result. The statistical descriptions of the manual GT measurement, deep learning model prediction (AI), and their difference (GT-AI) were displayed. For each maxillary basal bone index, the paired-sample Wilcoxon signed-rank test was performed between the AI and GT to investigate the prediction bias, owing to the lack of normality.

### Demographic parity-guided model ensemble

This study compared the GT and AI in different sex (male, female), age (18 to 29 years, 30 to 49 years, and >50 years), and tooth site (#11, #12, #21, #22) to find the sensitive attribute. Further, this study divided the overall CBCT dataset into 2 demographically different subsets, that is, the male-only dataset and female-only subsets. There are also training, validation, and test cohort (6:2:2 patient-wisely) within the 2 subsets. Then, 2 independent AI models were trained, validated, and tested on the male-only and female-only subsets, respectively. Finally, the prediction results of the 2 independent AI models on their respective test datasets were polled together to get the final ensembled test set performance to alleviate the population difference and increase the model robustness.

The multi-quantification performance of AI models was studied via a well-rounded series of evaluation metrics. Statistical descriptions of the GT, AI, and GT-AI were shown. The MAE and paired-sample Wilcoxon signed-rank test between the AI and GT were calculated. The ICC was used for determining the consistency between AI and GT. Likewise, the Pearson correlation coefficient (*r* value) was adopted for correlation estimation. From the qualitative view, this study turned to generate a visualization heatmap based on gradient-weighted class activation mapping (Grad-CAM) [30].

### Comparison between clinicians and AI in precision and efficacy

Clinicians with different background knowledge and clinical experience (junior and senior clinicians) were invited to manually measure basal bone indices on the 10% images randomly selected from the test set. The manual measurements by the 2 clinicians were compared to the GT and that of the predictions by ensembled AI models, and their corresponding time costs were recorded.

### Statistical analysis

The statistical analysis was performed using SPSS (26.0, IBM, USA). The continuous variables were presented in mean and standard deviation (SD). The prediction accuracy of the model was assessed using the paired-sample Wilcoxon signed-rank test, MAE, MSE, Pearson correlation coefficient, and ICC. The statistically significant level was set at α = 005.

## Data Availability

The data that support the findings of this study are available from the corresponding author (Zetao Chen) upon reasonable request and through collaborative investigations. The code is released at https://github.com/yoshua133/multi_quantification.
