# Supplementary material for: Multi-Quantifying Maxillofacial Traits via a Demographic Parity-Based AI Model
Source: BME Front. 2024 Aug 13;5:0054. doi: 10.34133/bmef.0054 (PMC11319927; doi:10.34133/bmef.0054)
Supplement: Supplementary 1 — Materials and Methods Table S1 Figs. S1 to S4 [file bmef.0054.f1.docx]

**Supplementary Materials**

**A Janus Adhesive Hydrogel with Integrated Attack and Defense for Bacteria Killing and Antifouling**

Kai Ren^1^, Xiang Ke^1^, Miao Zhang^1^, Yuan Ding^1^, Hao Wang^1^, Hong Chen^1^, Jing Xie*^1^ and Jianshu Li*^1, 2, 3^

^1^College of Polymer Science and Engineering, State Key Laboratory of Polymer Materials Engineering, Sichuan University, Chengdu 610065, P.R. China. ^2^State Key Laboratory of Oral Diseases, West Chin Hospital of Stomatology, Sichuan University, Chengdu 610041, P.R. China. ^3^Med-X Center for Materials, Sichuan University, Chengdu 610041, P.R. China.

*Corresponding authors: Jing Xie, Jianshu Li

E-mail: xiej@scu.edu.cn, jianshu_li@scu.edu.cn

*Corresponding author: Jing Xie, Jianshu Li

Tel: +86-28-85469978

Fax: +86-28-85405402

E-mail: xiej@scu.edu.cn, jianshu_li@scu.edu.cn

Supplementary experimental section

**Synthesis of** **poly ethylene glycol diacrylate (PEGDA) (1)**

Firstly, 20 g PEG (35 kDa) flakes and 160 μL TEA as deacid reagent were dissolved in 100 mL CH_2_Cl_2_, then 243 μL acryloylchloride was added to the mixed solution. The reaction mixture was stirred for 24 h at 25 ^o^C in darkness under nitrogen atmosphere to produce PEGDA via the esterification. When the reaction was finished, chloride acid was removed by adding 30 mL 2 M sodium carbonate solution. The separated organic phase dried by anhydrous MgSO_4_ was concentrated and volatilized the CH_2_Cl_2_ solvent in 45 ^o^C vacuum drying to obtain crude product. Finally, the crude product dissolved in deionized water was dialyzed for three days (3500 Da dialysis bag) and lyophilized to obtain PEGDA.


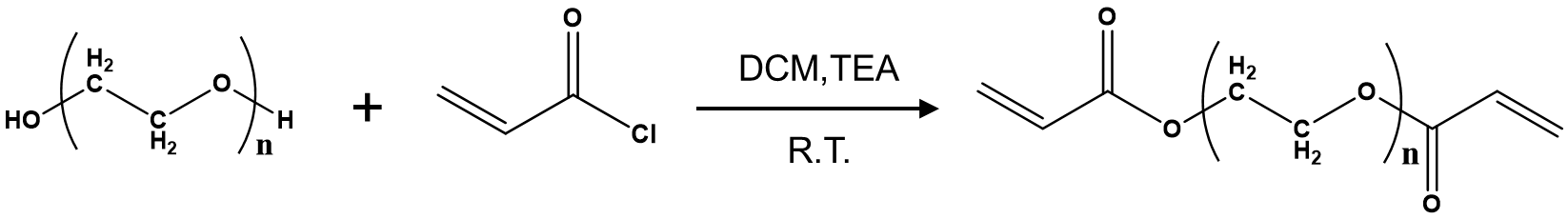


The synthesis of PEGDA (1).

**Synthesis of levodopa acrylamide (levadopaMA) (2)**

Besides, the synthesis of levodopaMA could be carried out by the amidation of levodopa and acryloylchloride. A method adopted by Ganesh et al. was properly modified here. 12.1 g Na_2_B_4_O_7_·10H_2_O and 5 g Na_2_CO_3_ were firstly dissolved in 480 mL deionized water that has been degassed by negative pressure, followed by nitrogen bubbling for 30 min to further remove the air. Then, 3.12 g levodopa was dissolved in the solution by stirring for 15 min under the protection of nitrogen. Next, 10 ml 50% v/v acryloylchloride THF solution was dropwise added to the aqueous solution within 30 min at 0 ^o^C. Finally, after 9 g Na_2_CO_3_ was added to the mixture to maintain the pH= 9.0, the reaction solution was stirred for 24 h at 25 ^o^C in darkness under nitrogen atmosphere to produce levodopaMA. When the reaction was finished, the resulting solution was acidified to pH = 2 with HCl solution and stirred slowly to release generated gas. The organic phase was extracted from the aqueous solution by EA and dried by MgSO_4_. The final levodopaMA was obtained by removing the EA solvent in 25 ^o^C vacuum drying.


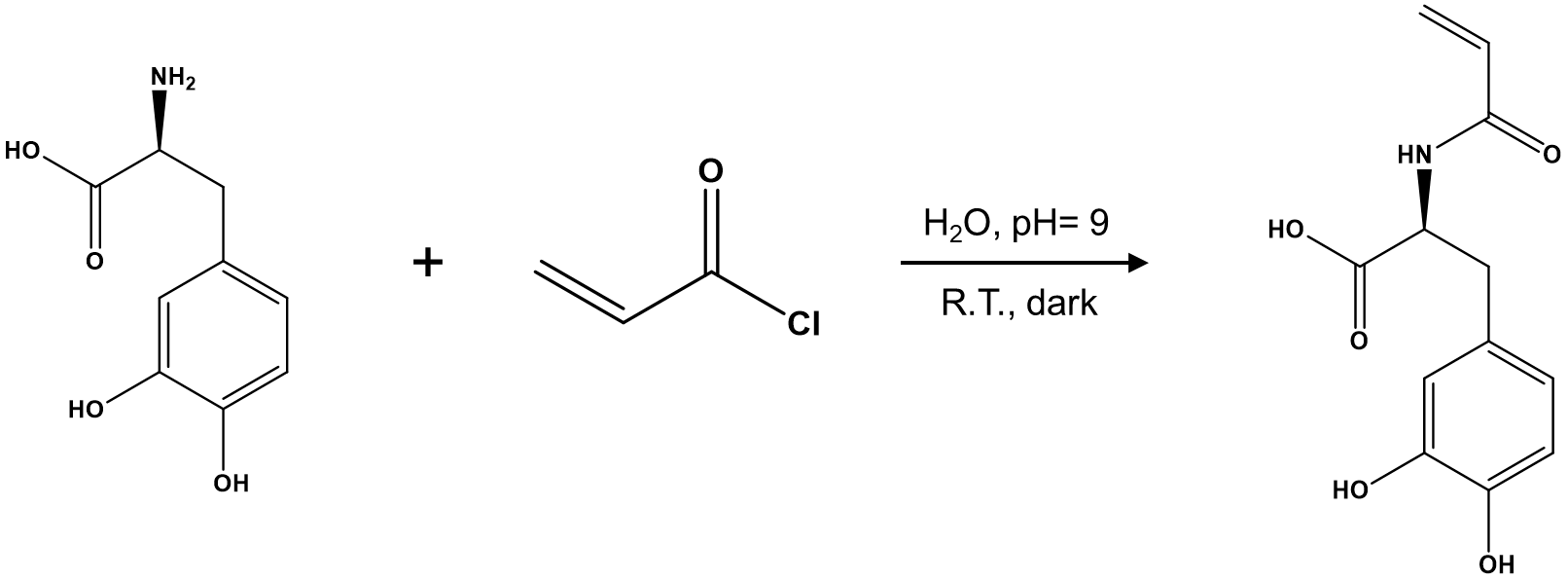


The synthesis of levadopaMA (2).

**Synthesis of poly N, N-dimethylacrylamide-co-levodopa acrylamide (DCL) copolymer (3)**

2.08 mL DMA, 388 mg levodopaMA and 40 mg AIBN were dissolved in 50 mL H_2_O/DMSO mixed solvent (compound ratio is 3:7) Then the mixture was bubbled with nitrogen for 30 min to remove the dissolved oxygen. Finally, the polymerization was conducted by continuously stirring the reaction solution at 60 ^o^C for 5 h under nitrogen atmosphere. When the reaction is finished, the resulting mixture was dialyzed for three days (3500 Da dialysis bag) and lyophilized to obtain DCL copolymer.


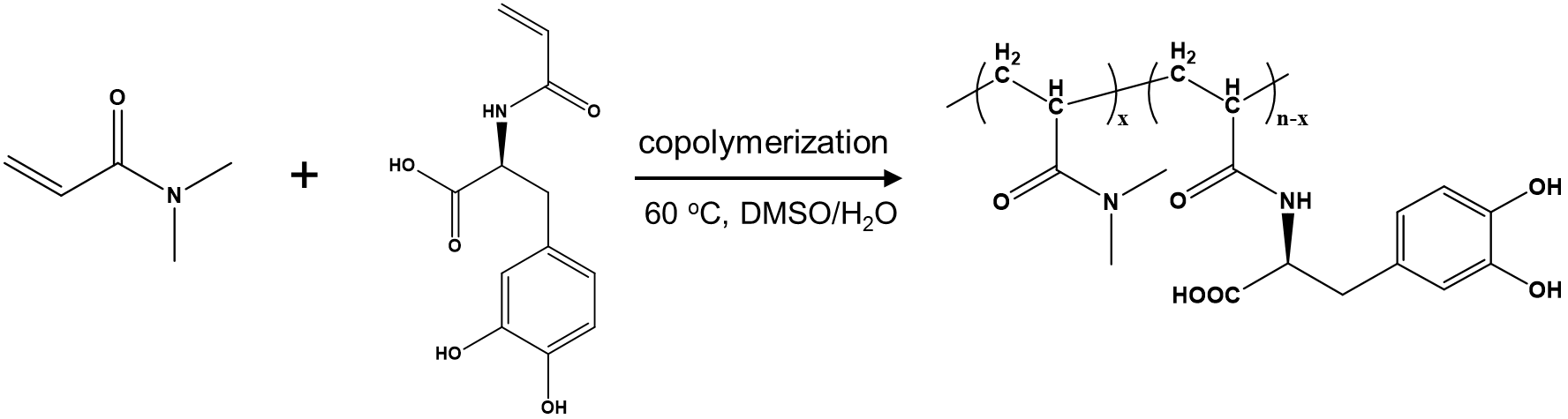


The synthesis of DCL copolymer (3).

**Supplementary data Section.**


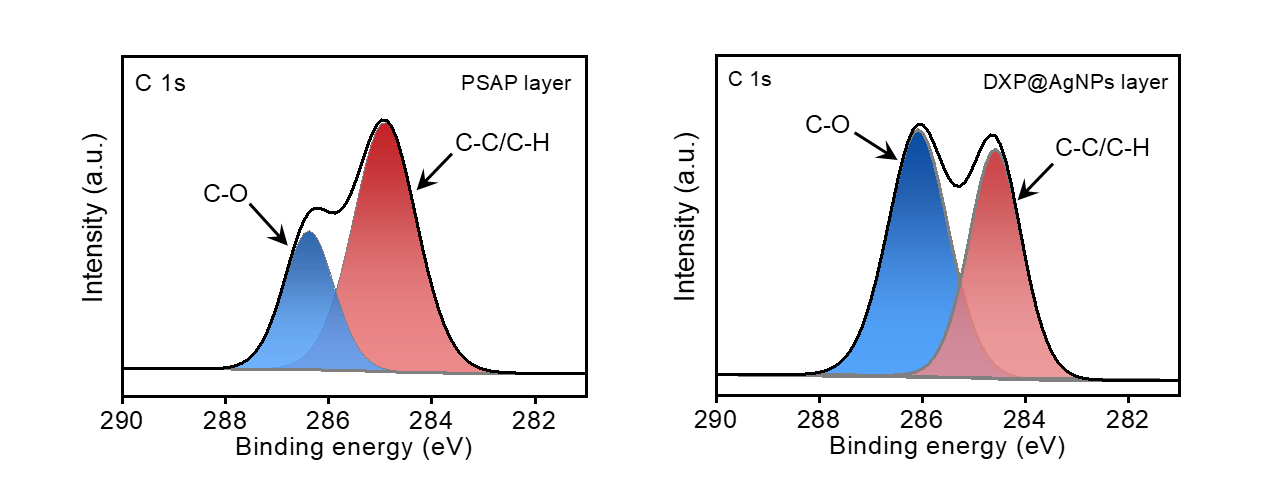


Figure S1. The XPS high-resolution C 1s spectra of PSAP layer and DXP@AgNPs layer for PSAP/DXP@AgNPs.


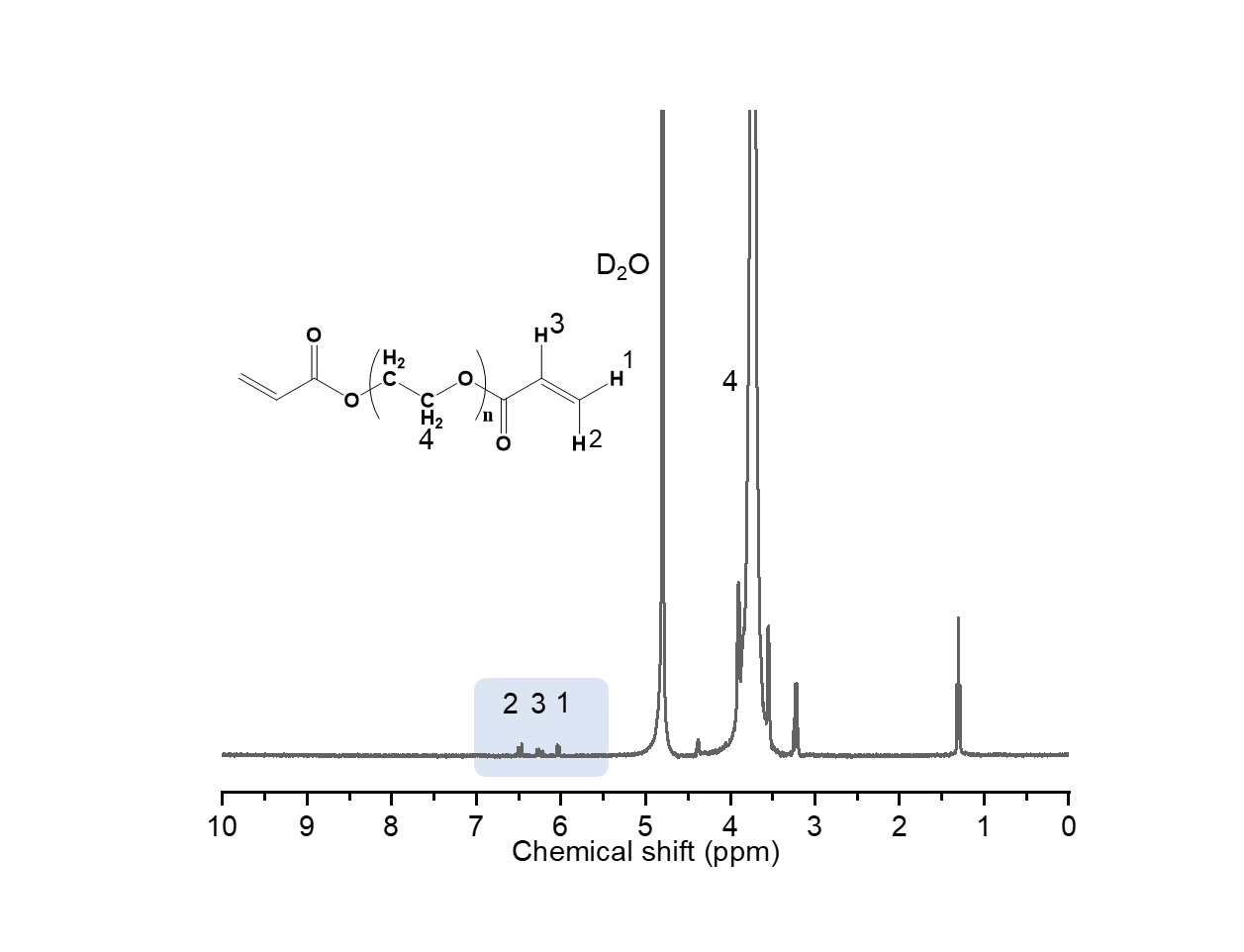


Figure S2. The ^1^H NMR spectrum of PEGDA


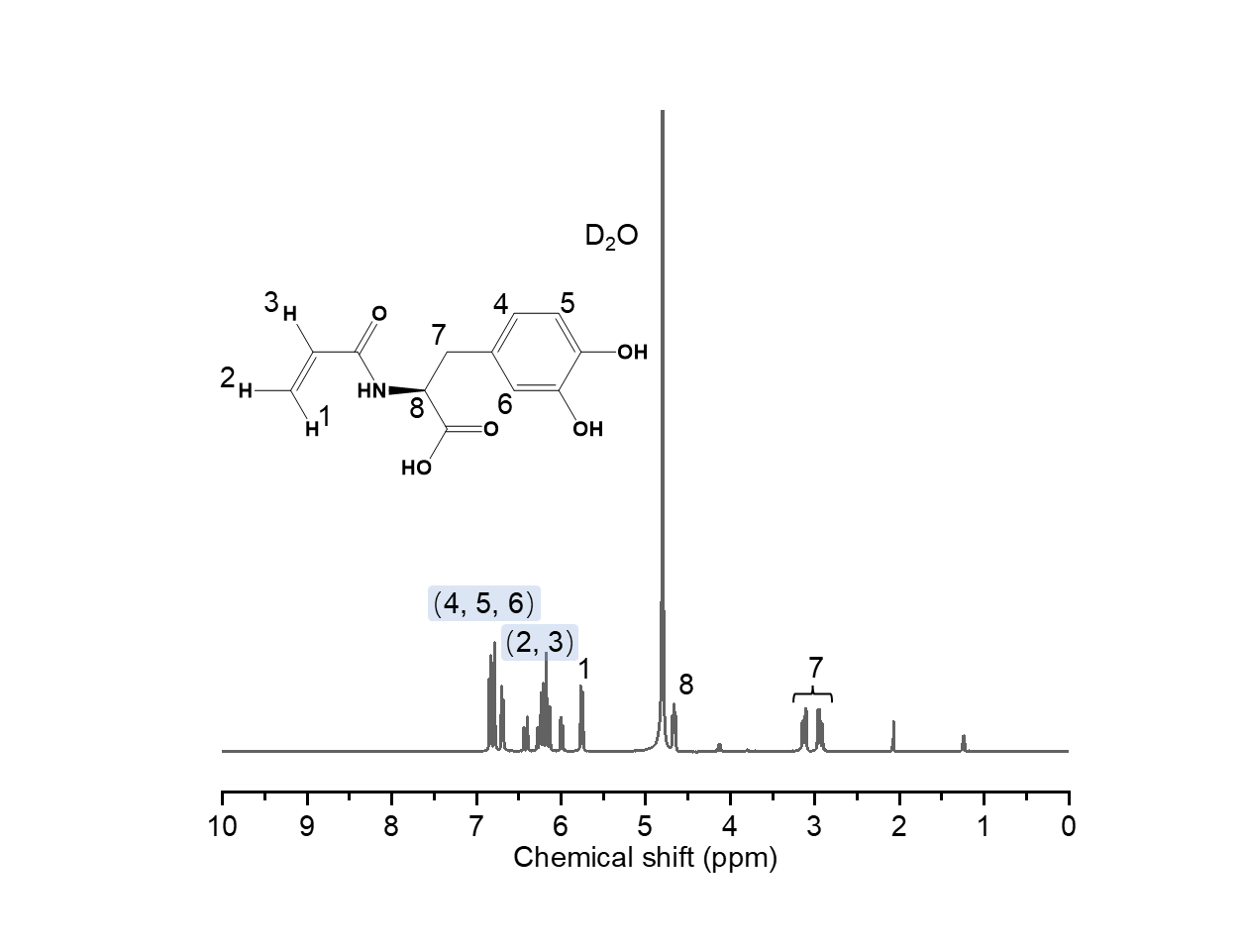


Figure S3. The ^1^H NMR spectrum of levodopaMA


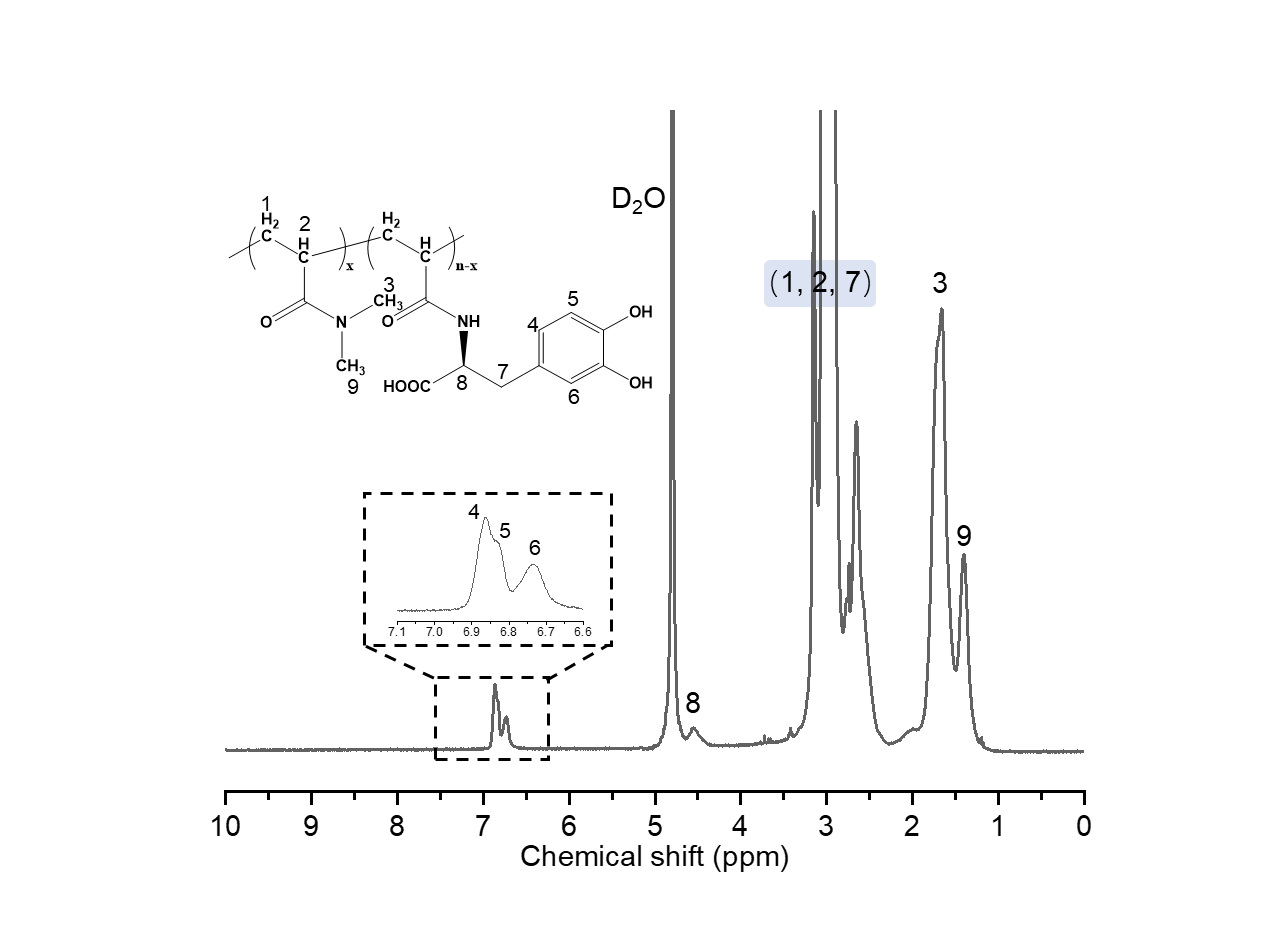


Figure S4. The ^1^H NMR spectrum of DCL copolymer.

Figure S5. The standard curve of BSA protein.

**Reference**

[1] Ganesh K, Jung J, Park JW, Kim BS, Seo S. Effect of substituents in mussel-inspired surface primers on their oxidation and priming efficiency. *ChemistryOpen*. 2021; 10(8): 852-859.
